# Creatine Fails to Augment the Benefits from Resistance Training in Patients with HIV Infection: A Randomized, Double-Blind, Placebo-Controlled Study

**DOI:** 10.1371/journal.pone.0004605

**Published:** 2009-02-26

**Authors:** Giorgos K. Sakkas, Kathleen Mulligan, Makani DaSilva, Julie W. Doyle, Hootan Khatami, Thomas Schleich, Jane A. Kent-Braun, Morris Schambelan

**Affiliations:** 1 Department of Medicine, University of California San Francisco, San Francisco, California, United States of America; 2 Division of Endocrinology, San Francisco General Hospital, San Francisco, California, United States of America; 3 Department of Medicine, University of Thessaly, Thessaly, Greece; 4 Northern California Institute for Research and Education, San Francisco, California, United States of America; 5 Department of Chemistry & Biochemistry, University of California Santa Cruz, Santa Cruz, California, United States of America; 6 Department of Exercise Science, University of Massachusetts, Amherst, Massachusetts, United States of America; McMaster University, Canada

## Abstract

**Background:**

Progressive resistance exercise training (PRT) improves physical functioning in patients with HIV infection. Creatine supplementation can augment the benefits derived from training in athletes and improve muscle function in patients with muscle wasting. The objective of this study was to determine whether creatine supplementation augments the effects of PRT on muscle strength, energetics, and body composition in HIV-infected patients.

**Methodology/Principal Findings:**

This is a randomized, double blind, placebo-controlled, clinical research center-based, outpatient study in San Francisco. 40 HIV–positive men (20 creatine, 20 placebo) enrolled in a 14-week study. Subjects were randomly assigned to receive creatine monohydrate or placebo for 14 weeks. Treatment began with a loading dose of 20 g/day or an equivalent number of placebo capsules for 5 days, followed by maintenance dosing of 4.8 g/day or placebo. Beginning at week 2 and continuing to week 14, all subjects underwent thrice-weekly supervised resistance exercise while continuing on the assigned study medication (with repeated 6-week cycles of loading and maintenance). The main outcome measurements included muscle strength (one repetition maximum), energetics (^31^P magnetic resonance spectroscopy), composition and size (magnetic resonance imaging), as well as total body composition (dual-energy X-ray absorptiometry). Thirty-three subjects completed the study (17 creatine, 16 placebo). Strength increased in all 8 muscle groups studied following PRT, but this increase was not augmented by creatine supplementation (average increase 44 vs. 42%, difference 2%, 95% CI −9.5% to 13.9%) in creatine and placebo, respectively). There were no differences between groups in changes in muscle energetics. Thigh muscle cross-sectional area increased following resistance exercise, with no additive effect of creatine. Lean body mass (LBM) increased to a significantly greater extent with creatine.

**Conclusions / Significance:**

Resistance exercise improved muscle size, strength and function in HIV-infected men. While creatine supplementation produced a greater increase in LBM, it did not augment the robust increase in strength derived from PRT.

**Trial Registration:**

ClinicalTrials.gov NCT00484627

## Introduction

In people with HIV infection and other chronic diseases, maintenance or augmentation of muscle mass is important for preserving functional status and forestalling disease progression [1]. Progressive resistance exercise training (PRT), either alone or in combination with aerobic exercise training, can increase muscle mass and improve physical performance in persons with HIV infection [2]–[10]. Many individuals employ so-called ergogenic aids, including substances such as anabolic steroids and growth hormone, to facilitate muscle accrual, and to enhance the anabolic effect of PRT on body composition with a view to both functional and aesthetic improvements [11], [12]. Creatine monohydrate is a nutritional supplement that has been shown to enhance short-term energy availability during intense exercise and to improve recovery between intense exercise bouts [13], [14]. Based on such findings, it is being used by competitive and recreational athletes as an ergogenic aid to improve their training efficiency. Although in some populations creatine supplementation has been shown to improve isotonic strength [15], torque [16] and power [17] other studies have failed to show any such effects [18]–[21].

To date, there have been no specific studies of the efficacy of the medical use of creatine supplementation in people with HIV infection. Further, the effects of creatine supplementation in combination with PRT on muscle energetics (e.g. mitochondrial function, resistance to fatigue, or intramuscular fat content) are not known in the HIV population, even though these are considered the primary benefits of creatine supplementation. The present study was designed to test the hypothesis that the medical use of creatine supplementation would augment increases in muscle strength derived from PRT in HIV-positive adults. The functional impact and potential mechanisms of these strength gains were also assessed.

## Methods

### Protocol

The protocol for this trial and supporting CONSORT checklist are available as supporting information; see Checklist S1 and Protocol S1.

### Ethics

The study was approved by the Committees on Human Research at the University of California San Francisco and the San Francisco VA Medical Center. All subjects gave written informed consent.

### Participants

**Figure 1:**
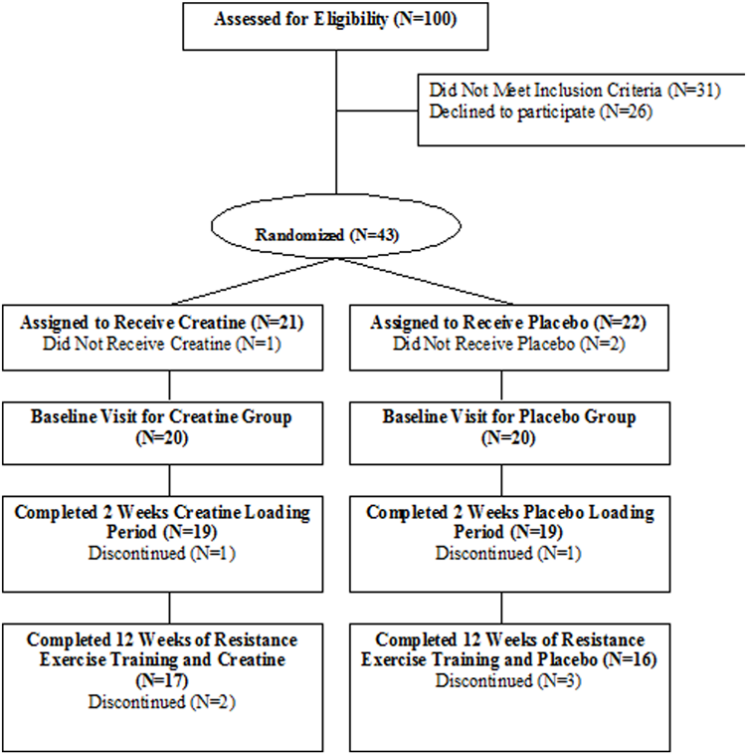
CONSORT Diagram showing the disposition of subjects randomized to receive creatine monohydrate or an equivalent number of placebo capsules for 14 weeks.

Forty-three clinically-stable, sedentary HIV-positive subjects (42 men, 1 woman) living in the San Francisco Bay area were recruited by our study coordinators and studied between August 2001 and January 2004 (Figure 1). Subjects using antiretroviral therapy (ART) were required to be on stable regimens for at least 30 days before enrollment and were asked to remain so during the study. Subjects who had received no ART for the preceding 30 days and had no plans to initiate therapy during the study were also eligible.

Exclusion criteria included regular resistance exercise training, use of anabolic hormones and other putative ergogenic aids (e.g. amino acids, protein supplements, β-agonists), serum creatinine >1.5 mg/dl; history of renal disease; creatine kinase (CK) >1.5 times the upper limit of normal (ULN); hemoglobin <8.5 g/dl; liver transaminase or lactate dehydrogenase levels ≥5×ULN; uncontrolled diarrhea, nausea, or vomiting; impaired oral food intake; untreated hypogonadism; pharmacologic use of anabolic or immune modulating therapies; systemic infection within 30 days; history of heart disease; and current pregnancy or lactation. Subjects on stable testosterone replacement for ≥6 months were eligible.

### Sample Size

Sample size calculations were based on other studies with similar design and allow for a detection of standardized effect sizes of 0.9 with 80% power to detect changes in intramuscular phosphocreatine (PCr) levels.

### Randomization

Subjects were randomized, in a blinded 1∶1 fashion, to receive identical-appearing capsules containing either pure creatine monohydrate or placebo (both provided by Jarrow Universal Herbs Inc., Union City, CA) for 14 weeks (Figure 1). A computer-generated randomization list was prepared by a statistical consultant and given directly to the research pharmacy at San Francisco General Hospital (SFGH), where the capsules were bottled and dispensed. All data were collected and analyzed without knowledge of treatment assignment.

Three subjects (1 creatine, 2 placebo), including the one woman, withdrew after randomization but before baseline testing or initiation of treatment and are thus excluded from the analysis.

### Intervention

Subjects began with a loading dose of 20 g/day (divided among four doses) or an equivalent number of placebo capsules for 5 days, followed by maintenance dosing of 4.8 g/day (in two 2.4 g doses) or placebo. Maintenance dosing was interrupted briefly for additional 5-day loading cycles at the beginning of weeks 7 and 13. This dosing regimen is similar to those employed in several recent studies (as reviewed by Nissen [13]). Subjects were given a non-caffeinated fruit juice beverage containing 20 g of simple carbohydrate to take with each dose of study medication [22].

#### Training Regimen

After two weeks, all subjects began a 12-week program of supervised PRT, while continuing on their assigned study medication. Training was performed using a Hoist 5000 Multi-Gym Fitness System (San Diego, CA). Subjects exercised for 1½ hours 3 days weekly for 12 weeks. The training included ankle dorsiflexion, ankle plantar flexion, ankle plantar flexion with bent knee, leg press, leg curls, pec decks chest exercises, tricep pushdown, bicep curl and three types of abdominal crunches. Strength in each muscle group was assessed by the one repetition maximum (1RM) test [23] except in the rectus abdominus, in which the number of sit-ups performed in 30 seconds was recorded. Each training session included 4 sets of 8 repetitions at 80% of the 1RM for each exercise. The 1RM for each exercise was reassessed once every two weeks and training intensity adjusted to maintain 80% of the 1RM. All measurements were performed after two days of rest in absence of any symptoms of muscle soreness or fatigue.

### Objectives

The primary aim was to compare the changes in muscle strength (1RM, sit-ups and MVC) from week 0 to week 14 in the two treatment groups. Secondary aims were to compare changes in muscle size, composition, energetics and fatigue, as well as body composition and biochemistry.

### Outcome Measurements

Subjects were studied in the General Clinical Research Center (GCRC) at SFGH and in the Magnetic Resonance Unit at the San Francisco VA Medical Center. Height and weight were recorded with subjects wearing a hospital gown. Measurements of muscle strength, size, composition, energetics and fatigue, as well as body weight and composition and serum biochemistries, were made at baseline, after two weeks of treatment with creatine or placebo (before PRT began), and again after 12 weeks of PRT (study week 14). Safety was monitored throughout the study.

#### Muscle Energetics and Fatigue

Non-invasive measures of intracellular phosphocreatine (PCr), inorganic phosphorous (Pi), and pH were obtained from the dorsiflexor muscle of the right ankle by ^31^P magnetic resonance spectroscopy (MRS) using a 30-cm bore 1.9 T superconducting Oxford magnet, as described in detail elsewhere [24], [25]. Subjects performed two contraction protocols on the same day, separated by 20 minutes of rest. The first protocol consisted of a 15-second maximum voluntary contraction (MVC), which was used to determine the muscle's capacity for oxidative phosphorylation [24] reported as the half-time (T_1/2_) of PCr recovery following the contraction. The recovery analysis indicated that our MRS system can detect PCr changes of 0.5 mM. The second protocol was used to assess muscle function during high-intensity, fatiguing conditions. Muscle force and energetics were measured simultaneously during 36 consecutive MVCs over 6 minutes, one every 10 seconds. The fatigue profile was calculated as described by Karatzaferi et al [26].

#### Dorsiflexor Strength

Isometric MVC force was recorded during ankle dorsiflexion while the leg was positioned in the magnet for the MRS studies [24], [25] The highest force from the 3 MVCs was used to quantify dorsiflexion strength [24] and scale performance during the aforementioned fatigue task.

#### Muscle Size and Composition

Proton T_1_-weighted magnetic resonance imaging (MRI) was used to visualize the cross-sectional area (CSA) of the thigh and calf using a 1.5 T whole body Siemens Magnetom Vision System, as described previously [24]. Data were analyzed using a customized software program (Interactive Data Language Research Systems, Inc., Boulder, CO). The coefficient of variation of muscle CSA measurements was 0.6%.

#### Body Composition

Whole body and regional fat and lean body mass (LBM) were measured using a Lunar model DPX dual energy x-ray absorptiometer (DEXA) (Madison, WI) [27], [28].

#### Biochemical Measurements

Fasting blood samples were collected for determination of plasma creatine and lactate and serum creatinine, creatine kinase (CK), lipid, glucose, and insulin levels. Plasma creatine was determined by liquid chromatography – tandem mass spectrometry (LC/MS) using a Surveyor LC interfaced with a TSQ Quantum Ultra triple-stage quadrupole MS (Thermo-Finnigan, San Jose, CA) operated in the positive ion mode using atmospheric pressure chemical ionisation. Quantitation was achieved using selected reaction monitoring of the transitions m/z 114 to m/z 44 for creatine and m/z 117 to m/z 47 for the internal standard. The lower limit of quantitation was 2 µg/mL (18 µmol/L). Intra-day precision ranged from 3.2% to 8.9%, and recovery from 100.3% to 103.4%. Plasma lactate was measured using a YSI STAT2300 analyzer (Yellow Springs, OH), and serum insulin by radioimmunoassay (Linco Research Inc., St. Charles, MO) in the GCRC Core Laboratory. The remaining assays were performed in the SFGH Clinical Laboratories. Insulin resistance was calculated by homeostasis model assessment (HOMA-IR [29]).

#### Safety

Serum creatinine and CK levels were monitored biweekly. Study medication was suspended for one week if creatinine levels increased to >1.8 mg/dl or CK to >450 U/liter. If, after one week, creatinine and/or CK levels returned to baseline levels, study medication was resumed at half of the initial dosing level and creatinine and/or CK levels were monitored weekly. If creatinine and/or CK levels again exceeded the aforementioned values, study drug was discontinued but subjects continued on study (including exercise). If subjects complained of soreness, the workload for the affected muscle group(s) was reduced until the soreness resolved.

### Statistical Analysis

All safety analyses were performed on an intent-to-treat basis in all 40 subjects who underwent baseline testing. Efficacy analyses were performed in the evaluable subset, defined as the number of subjects assigned to each treatment in whom paired results (baseline to week 2, baseline to week 14) were available. The numbers of subjects in whom these results are available are indicated in each table.

Within-group changes from baseline to week 2 and baseline to week 14 were evaluated using paired t-tests. Absolute changes (week 0 to week 2 and week 0 to week 14) in the creatine group were compared with those in the placebo group using unpaired t-tests, and the 95% confidence interval for the differences between mean changes calculated. All statistical analyses were performed using Statview (SAS Institute Inc. Cary, NC). Data are the mean and (SD). Two-tailed p-values <0.05 were considered statistically significant. Non-parametric analyses were also performed and yielded similar results.

## Results

### Subjects

Of the 40 subjects (20 creatine, 20 placebo) who underwent baseline testing and received at least one dose of study medication, 25% were African-American, 15% Hispanic, and 60% Caucasian. There were no differences between groups in age, racial distribution, BMI, CD4 count, years of known HIV infection, or in the classes of ART used (Table 1).

**Table 1:** Baseline Characteristics of the Study Population.

	Creatine	Placebo
N	20	20
Age (yrs)	44 (9)	44 (8)
BMI (kg/m^2^)	23.7 (2.6)	23.7 (2.5)
Total body fat (%)	18.4 (6.7)	16.0 (5.3)
Testosterone (ng/dl)	725 (295)	597 (217)
Yrs since HIV diagnosis	10 (7)	10 (5)
CD4 (cells/µl)	448 (310)	460 (278)
**Current antiretroviral therapy:**		
PI (%)	40	35
NRTI (%)	80	75
NNRTI (%)	30	30
No Antiretroviral (%)	20	35

Subject characteristics at baseline according to the assigned study medication. Data are mean (SD).

**Abbreviations:** PI, protease inhibitor; NRTI, nucleoside analogue reverse transcriptase inhibitors; NNRTI, non-nucleoside analogue reverse transcriptase inhibitors.

### Follow-up and Adherence to Study Treatments

Thirty-three subjects (83%) completed the study. Seven subjects (3 creatine, 4 placebo) withdrew for the following reasons: hypersensitivity reaction (1 creatine), schedule conflicts (2 creatine), family emergency (1 placebo), and non-adherence (missing three consecutive PRT sessions [3 placebo]). Subjects who completed the study attended 95% of their training sessions. Adherence to the treatment regimen was evidenced by the fact that plasma creatine levels increased substantially in all subjects who were receiving creatine at week 14 (see below).

### Effects on muscle strength

**Figure 2:**
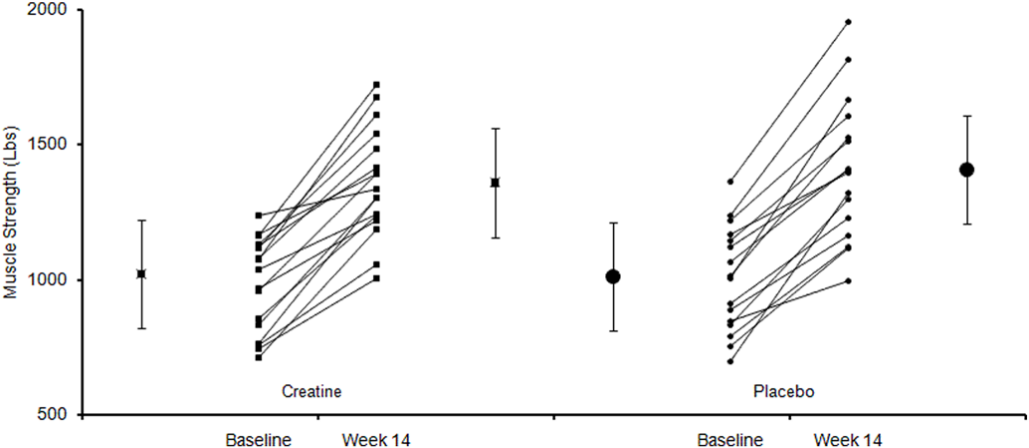
Muscle strength before and after 14 weeks of study. Large rectangles represent the average values, while smaller rectangles individual data before and after creatine supplementation. Similarly, large circles represent the average values, while smaller circles individual data before and after placebo supplementation. Data are the sum of strength (1 RM) in 8 muscle groups in subjects randomized to receive creatine monohydrate or placebo. Strength increased significantly within each treatment group (P<0.01), but there was no significant difference between groups in the magnitude of the increase (average increase 44 and 42% (difference 2%, 95% CI −9.5% to 13.9%) in creatine and placebo, respectively, P = 0.58).

At baseline, all measures of strength were similar in the two study groups (Table 2). At week 14, average 1RM strength increased in all muscle groups (Table 2, Fig. 2). Contrary to our hypothesis, the magnitude of the increase in strength was not greater with creatine (average increase 44% for creatine and 42% for placebo, difference 2%, 95% CI −9.5% to 13.9%; P = 0.58). In addition, the rate of strength gain did not differ between groups. Dorsiflexor muscle isometric strength (MVC) did not change significantly in either group (Table 2).

**Table 2:** Muscle Strength and Energetics at Baseline and Changes on Study.

	Baseline		Changes wk 0 to wk 2		Changes wk 0 to wk 14		Differences between groups Mean changes (95% CI)	
	Creatine	Placebo	Creatine	Placebo	Creatine	Placebo	Wk 0 to Wk 2	Wk 0 to Wk 14
**Strength in individual muscle groups:**								
N	20	20	19	19	17	16	N/A	N/A
Ankle Dorsiflexion (lbs)	90 (21)	101 (21)	5 (8)	6 (12)	60 (26)*	59 (27)*	−1 (8 to 5)	1 (−18 to 20)
Ankle Plantar Flexion (lbs)	176 (44)	174 (37)	15 (21)	6 (17)	80 (39)*	68 (54)*	9 (−3 to 22)	12 (−22 to 45)
Ankle Plantar Flexion Bent Knee (lbs)	207 (71)	176 (56)	10 (19)	23 (29)	64 (46)*	106 (67)*	−13 (−29 to 3)	−42 (−83 to 1)
Leg Presses (lbs)	248 (55)	245 (54)	2 (17)	14 (29)	66 (56)*	75 (49)*	−12 (−28 to 4)	−9 (−47 to 27)
Leg Curls (lbs)	106 (26)	113 (22)	6 (10)	4 (14)	43 (22)*	38 (27)*	3 (−5 to 11)	5 (−13 to 22)
Pec Deck (lbs)	88 (23)	94 (26)	3 (9)	3 (6)	35 (15)*	32 (14)*	0 (−5 to 4)	2 (−8 to 12)
Biceps Pushdown (lbs)	51 (14)	51 (12)	1 (4)	1 (6)	14 (10)*	12 (9)*	0 (−4 to 4)	2 (−5 to 9)
Triceps Curls (lbs)	53 (10)	54 (11)	2 (3)	1 (6)	16 (8)*	14 (8)*	1 (−2 to 4)	2 (−4 to 7)
Abdominal Crunches (rep)	19 (5)	17 (5)	1 (2)	1 (3)	8 (4)*	9 (5)*	0 (−1 to 2)	−1 (−4 to 2)
MVC (N)	241 (53)	240 (61)	−1 (67)	27 (51)	9 (38)	39 (50)	−28 (−76 to 9)	−30 (−65 to 5)
Lower Leg Flexion	(N = 16)	(N = 18)	(N = 12)	(N = 14)	(N = 13)	(N = 14)		
**Muscle function (^31^P MRS): 15-second maximal voluntary contraction (MVC) protocol:**								
**N**	17	17	13	12	14	13	N/A	N/A
At rest (before MVC):								
PCr (mM)	37 (2)	36 (3)	−1 (3)	0 (3)	0 (3)	0 (2)	−1 (−3 to 1)	0 (−2 to 2)
Pi (mM)	5.8 (2.3)	6.1 (2.6)	0.7 (2.9)	−0.4 (2.6)	0.2 (2.9)	0.2 (2.4)	1.1 (−1.2 to 3.5)	0.1 (−2.0 to 2.2)
PCr/Pi	7.6 (3.7)	7.3 (3.6)	−0.3 (6.1)	0.4 (4.8)	0.5 (6.8)	−1.1 (4.0)	−0.7 (−5.3 to 3.8)	1.6 (−2.9 to 6.1)
pH	7.15(0.06)	7.12(0.05)	−0.04(0.07)	−0.02(0.06)	−0.03(0.06)	−0.02(0.05)	−0.02(−0.08 to 0.03)	−0.01(−0.06 to 0.03)
Immediately following a 15-sec MVC:								
PCr (mM)	18 (4)	16 (5)	−2 (6)	−1 (5)	−2 (5)	−1 (8)	−1 (−5 to 4)	−1 (−6 to 5)
% of resting PCr	48 (11)	44 (13)	−4 (15)	−4 (10)	−4 (12)	−3 (21)	0 (−11 to 10)	−1 (−14 to 13)
Pi (mM)	23 (6)	25 (6)	2 (8)	2 (4)	−2 (6)	0 (8)	0 (−5 to 6)	−2 (−8 to 4)
PCr/Pi	0.9 (0.5)	0.7 (0.3)	0.1 (0.5)	−0.1 (0.3)	0.0 (0.5)	0.0 (0.5)	0.0 (−0.4 to 0.3)	0.0 (−0.4 to 0.4)
pH	7.09 (0.23)	7.06 (0.13)	−0.11 (0.26)	0.03 (0.20)	−0.02 (0.38)	0.01 (0.13)	−0.14(−0.34 to 0.05)	−0.03(−0.26 to 0.20)
PCr recovery (t_1/2_, sec)	30 (9)	29 (12)	−2 (12)	−1 (15)	−4 (9)	−4 (16)	−1 (−12 to 11)	0 (−10 to 11)
**Muscle function (^31^P MRS): 6 minute exercise protocol:**								
N	15	15	10	10	12	10	N/A	N/A
At rest :								
PCr (mM)	37 (3)	37 (2)	−1 (3)	−1 (3)	0 (2)	0 (3)	0 (−3 to 3)	0 (−2 to 3)
Pi (mM)	5.4 (2.6)	5.1 (2.4)	0.9 (3.5)	0.7 (2.9)	−0.4 (1.9)	−0.1 (3.1)	0.1 (−2.8 to 3.1)	−0.3 (−2.6 to 2.0)
PCr/Pi	10.0 (8.0)	9.6 (5.9)	−2.7 (7.6)	−2.1 (4.3)	−0.9 (7.6)	0.9 (6.5)	−0.6 (−6.4 to 5.2)	−1.8 (−8.2 to 4.6)
pH	7.13(0.08)	7.09(0.06)	−0.03(0.08)	0.02(0.10)	0.02(0.09)	0.03(0.09)	−0.05(−0.13 to 0.04)	−0.01(−0.09 to 0.07)
Immediately following the 6-minute exercise protocol:								
PCr (mM)	8 (7)	7 (4)	2 (6)	−1 (4)	−1 (6)	−1 (5)	3 (−2 to 7)	0 (−5 to 5)
% of resting PCr	23 (20)	19 (11)	5 (16)	−3 (10)	−2 (15)	−2 (14)	8 (−5 to 20)	0 (−13 to 13)
Pi (mM)	33 (8)	35 (5)	0 (8)	2 (4)	1 (8)	1 (7)	−2 (−8 to 4)	0 (−7 to 7)
PCr/Pi	0.4 (0.5)	0.2 (0.2)	0.1 (0.3)	−0.1(0.1)	0.0 (0.4)	0.0 (0.2)	0.2 (0.0 to 0.4)	0.0 (−0.3 to 0.4)
pH	6.67(0.16)	6.67(0.25)	−0.06 (0.18)	0.04(0.12)	−0.03 (0.21)	0.07 (0.13)	−0.02(−0.16 to 0.13)	−0.10(−0.26 to 0.06)
PCr recovery (t_1/2_, sec)	36 (16)	46 (18)	−1 (28)	−2 (15)	−1 (17)	−10 (20)	1 (−21 to 22)	9 (−7 to 25)

Data are mean (SD). The mean differences and the 95% confidence intervals between treatment groups of the changes from baseline at weeks 2 and 14 were determined by unpaired t-tests. Asterisks (*) are used to indicate within-group changes from baseline that are statistically significant by paired t-test (P<0.05).

Strength was measured as the 1 repetition maximum (1RM) for each muscle group (in pounds) except for the rectus abdominus, in which strength was assessed as the number of sit-ups completed in 30 seconds; and maximum voluntary contraction (MVC, in Newtons) of the tibialis anterior muscle, which was assessed during dorsiflexion of the right foot. Muscle function was evaluated using two exercise protocols that were performed inside a 1.9T magnet. The first protocol consisted of a 15-second maximum voluntary contraction (MVC) that was used to assess the rate of PCr recovery (an index of oxidative capacity). The second protocol consisted of 36 consecutive MVCs, one every 10 seconds, with cycles of 6 seconds of contraction and 4 seconds of relaxation. This latter protocol was used to assess muscle fatigue.

**Abbreviations:** Rep, Repetitions; PCr, phosphocreatine; Pi, inorganic phosphate; PCr recovery (t _½_), time needed to recover half of the baseline PCr value.

### Effects on muscle energetics and fatigue

All measures of muscle energetics, including muscle oxidative capacity, were similar across groups at all time points, and were not significantly affected by creatine supplementation (Table 2). Likewise, the fatigue profile (the rate of force decline during the 6-minute exercise protocol) did not differ between or within the two groups at any time point (data not shown). Among subjects randomized to creatine, there was a weak association between changes in plasma creatine levels and changes in resting PCr (r = 0.53, P = 0.09) and no relationship with post-exercise PCr. In the two treatment groups combined, PCr recovery following 15-sec MVC improved significantly after PRT (T_1/2_ = −3.89 [−0.04 to −7.74] seconds, P = 0.047).

### Effects on body composition

Body weight and composition were similar at baseline (Table 3). By week 14, LBM had increased in both groups, with a greater increase in the creatine group (P = 0.01). Body weight increased only in the creatine group. No differences between groups were noted in total, trunk or limb fat content at any time point.

**Table 3:** Body Composition at Baseline and Changes on Study.

	Baseline		Changes wk 0 to wk 2		Changes wk 0 to wk 14		Differences between groups Mean changes (95% CI)	
	Creatine	Placebo	Creatine	Placebo	Creatine	Placebo	Wk 0 to Wk 2	Wk 0 to Wk 14
N	20	20	19	19	17	16	N/A	N/A
**DEXA Results (kg)**								
Lean body mass	56.0 (7.3)	57.4 (6.6)	0.9 (1.9)	0.4 (1.2)	2.3 (1.4)*	0.9 (1.4)*	0.5 (−0.5 to 1.5)	**1.4 (0.3 to 2.4)**
Limb (arm+leg) LBM	25.5 (3.8)	26.2 (3.4)	0.6 (0.9)*	−0.0 (0.7)	1.1 (0.9)*	0.7 (1.1)*	**0.7 (0.1 to 1.3)**	0.5 (−0.3 to 1.2)
Total body fat	13.7 (6.4)	11.8 (4.8)	0.2 (0.8)	0.2 (0.7)	0.3 (1.8)	0.4 (2.0)	−0.0 (−0.5 to 0.4)	−0.0 (−1.4 to 1.3)
Trunk fat	8.2 (4.0)	6.7 (3.1)	0.0 (0.5)	0.1 (0.5)	0.0 (1.2)	0.3 (1.5)	−0.2 (−0.5 to 0.1)	−0.3 (−1.2 to 0.7)
Limb (arm+leg) fat	4.8 (2.2)	4.5 (1.8)	0.2 (0.4)	0.0 (0.3)	0.2 (0.6)	0.0 (0.6)	0.2 (−0.1 to 0.4)	0.2 (−0.2 to 0.6)
**MRI Results**								
Thigh muscle CSA (cm^2^)	136.7 (24.0)	142.9 (18.3)	4.3 (4.8)	0.7 (4.8)	12.2 (7.8)*	9.3 (8.1)*	3.6 (−0.0 to 7.3)	2.9 (−3.3 to 9.1)
Thigh % EMCL	4.2 (1.3)	4.9 (2.1)	−0.1 (1.4)	−0.6 (1.6)	−0.1 (1.5)	−1.0 (2.3)	0.6 (−0.6 to 1.7)	0.9 (−0.6 to 2.4)
Thigh SAT area (cm^2^)	36.2 (18.7)	38.6 (20.3)	−0.8 (2.9)	2.4 (13.5)	−0.5 (4.1)	4.7 (14.6)	−3.2 (−10.3 to 3.9)	−5.2 (−13.1 to 2.7)
Calf muscle CSA (cm^2^)	74.7 (12.7)	73.9 (13.3)	0.2 (2.6)	−0.1 (2.8)	1.5 (3.8)	0.1 (3.5)	0.3 (−1.7 to 2.2)	1.4 (−1.4 to 4.1)
Calf % EMCL	4.7 (1.6)	4.6 (1.9)	0.8 (1.7)	0.5 (1.9)	1.1 (1.9)	0.9 (2.0)	0.3 (−1.0 to 1.6)	0.2 (−1.2 to 1.7)
Calf SAT area (cm^2^)	8.2 (5.6)	7.6 (5.4)	−0.3 (1.6)	0.6 (2.1)	0.2 (1.3)	0.4 (0.7)	−0.9 (−2.2 to 0.4)	−0.2 (−1.0 to 0.6)

Data are mean (SD). Limb fat is the sum of fat in the arms and legs. The mean differences and 95% confidence intervals between treatment groups of the changes from baseline at weeks 2 and 14 were determined by unpaired t-tests. Differences between groups in changes from baseline that are statistically significant (P<0.05) are highlighted in bold. Asterisks (*) are used to indicate within-group changes from baseline that are statistically significant by paired t-test (P<0.05).

**Abbreviations**: DEXA, dual energy X-ray absorptiometry; MRI, magnetic resonance imaging; CSA, cross-sectional area; SAT, subcutaneous adipose tissue; EMCL, extramyocellular fat (fat infiltration).

Thigh muscle CSA increased in both groups at week 14, but the magnitude of the increase did not differ significantly between groups. There were no changes in calf CSA in either group. Likewise, no changes in muscle or subcutaneous fat were noted at any time point in either treatment group.

### Biochemical and safety measures

There were no significant differences between groups in biochemical parameters at baseline (Table 4). Average plasma creatine levels increased significantly in the creatine group, with no change in those on placebo (P≤0.001 vs. placebo). Serum creatinine concentrations increased more with creatine than placebo (P = 0.001 and 0.002 at weeks 2 and 14, respectively). Triglycerides increased with creatine at week 14 (P = 0.04 vs. placebo). Total cholesterol decreased significantly in the placebo group at week 14, but the difference between groups was not statistically significant. At week 2, both insulin and HOMA-IR decreased transiently in the creatine group (P = 0.04 and 0.05 vs. placebo, respectively). There were no differences in lactate, glucose or CK levels between or within groups at any time point.

**Table 4:** Safety and Biochemical Measurements at Baseline and Changes on Study.

	Baseline		Changes wk 0 to wk 2		Changes wk 0 to wk 14		Differences between groups Mean changes (95% CI)	
	Creatine	Placebo	Creatine	Placebo	Creatine	Placebo	Wk 0 to Wk 2	Wk 0 to Wk 14
N	20	20	19	19	17	16	N/A	N/A
Plasma creatine (µg/mL)	4.6 (1.4)	4.6 (2.2)	26.0 (25.5)*	−0.4 (1.4)	22.1 (18.0)*	−1.4 (1.4)*	**26.4 (12.9 to 39.8)**	**23.5 (13.6 to 33.4)**
Serum creatinine (mg/dL)	1.0 (0.2)	1.0 (0.1)	0.2 (0.2)	0.0 (0.1)	0.2 (0.2)	0.1 (0.1)	**0.2 (0.1 to 0.3)**	**0.2 (0.1 to 0.3)**
Creatine kinase (U/L)	111 (51)	147 (75)	20 (57)	−15 (84)	20 (61)	8 (64)	35 (−13 to 84)	12 (−33 to 57)
Triglycerides (mg/dL)	169 (89)	222 (199)	91 (362)	65 (199)	78 (165)	−18 (75)	26 (−166 to 218)	96 (4 to 188)
Cholesterol (mg/dL)	183 (47)	185 (46)	1 (19)	−4 (23)	−6 (22)	−10 (18)*	4 (−10 to 18)	4 (−1 to 19)
Lactate (mmol/L)	1.4 (0.7)	1.1 (0.4)	0.0 (0.9)	0.0 (0.5)	0.0 (1.1)	−0.1 (0.7)	−0.1 (−0.5 to 0.4)	−0.1 (−0.8 to 0.6)
Glucose (mg/dL)	94 (12)	90 (9)	−2 (12)	−1 (9)	−6 (14)	0 (8)	−1 (−8 to 6)	−6 (−14 to 3)
Insulin (µIU/mL)	15.9 (5.9)	14.3 (5.2)	−1.6 (6.1)	3.0 (6.6)	1.2 (10.5)	2.7 (7.3)	**−4.6 (−9.1 to −0.2)**	−1.5 (−8.1 to 5.1)
HOMA-IR	3.6 (1.2)	3.3 (1.5)	−0.5 (1.5)	0.6 (1.5)	0.2 (2.5)	0.6 (1.7)	−1.0 (−2.0 to 0.0)	−0.4 (−2.0 to 1.2)

Data are the mean (SD). All measurements were performed under fasting conditions. The significance of differences between treatment groups in the changes from baseline at weeks 2 and 14 was determined by unpaired t-tests, and differences that are statistically significant (P<0.05) are highlighted in bold. Asterisks (*) are used to indicate within-group changes from baseline that are statistically significant by paired t-test (P<0.05).

Abbreviations: HOMA-IR: Homeostasis model assessment of insulin resistance (20).

To convert to SI units: creatine µg/mL to µmol/L multiply by 7.625; creatinine mg/dL to µmol/L multiply by 88.4; triglycerides mg/dL to mmol/L multiply by 0.01129; cholesterol mg/dL to mmol/L multiply by 0.02586; glucose mg/dL to mmol/L multiply by 0.05551; insulin µIU/mL to pmol/L multiply by 7.175.

With regard to the pre-defined toxicity management guidelines, 4 subjects, all in the creatine group, experienced increases in serum creatinine levels to >1.8 mg/dL. In all four cases, creatinine levels had returned to <1.8 mg/dL at week 14. CK levels increased to >450 U/liter 18 times in 14 subjects (10 on placebo and 4 on creatine). The increases were transient and likely related to exercise, and all had resolved by week 14.

## Discussion

In the present study, we found that PRT consistently increased muscle strength in HIV-infected men (Fig. 2). Although subjects receiving creatine supplementation had a greater increase in LBM than those on placebo, our results provided no evidence that creatine augmented the increase in strength derived from PRT. Thus, the increase in LBM in the creatine group was of no measurable functional benefit. Moreover, using ^31^P-MRS, we found no difference between groups in skeletal muscle energy metabolism at rest, during or following muscle contractions, or in the fatigue profile. Overall, these data indicate that PRT is a highly effective means of increasing strength in individuals with HIV infection, but supplementation with creatine, though safe, confers no additional increase in strength in this population.

Other studies of creatine supplementation have provided mixed results. In studies of healthy volunteers, short-term creatine supplementation (5–30 days) increased muscle size [30] and indices of short-term high intensity exercise performance, including muscle strength and power output [31], [32] as well as intramuscular PCr levels assessed by both ^31^P-MRS [33] and biopsy [34]. Chronic use of creatine (up to 12 months) has been associated with increases in total body weight [35], [36]. In contrast, other studies have failed to demonstrate a positive effect of creatine supplementation on strength, body composition or muscle energy metabolism [18]–[21]. The responses to creatine supplementation in patients with chronic diseases have been similarly mixed, with some studies showing positive effects [37]–[42] and others showing no benefit [43]–[46].

The pre-treatment PCr concentrations observed in the current study are consistent with those in the same muscle in healthy young and older adults [24], suggesting that no deficiency of PCr existed in this group of HIV-positive subjects. Although other studies have shown that creatine supplementation increased PCr levels in healthy adults with normal baseline levels [47] we saw no such increase in our subjects with HIV infection. We followed a protocol for creatine loading and maintenance that had been successfully applied in previous studies [13]. In view of the potential role of carbohydrate availability and insulin in stimulating the transport of creatine into muscle cells [22], we provided subjects with beverages containing 20 g of carbohydrate for consumption with each dose of study medication. Importantly, we raised plasma creatine to levels seen in previous studies [48]. Nonetheless, we saw no change in intramuscular PCr concentration at rest or following exercise. It is possible that factors specific to HIV infection or its therapies may account for this failure. For example, some HIV protease inhibitors inhibit glucose uptake by skeletal muscle [49], [50] and might have also interfered with creatine uptake into muscle. In addition use of another antiretroviral agent, zidovudine, has been associated with an accelerated rate of depletion of PCr during exercise in HIV-infected subjects [51] and may have had a similar effect in our population. An assessment of the effects of specific antiretroviral medications on outcome measures was not possible in view of the relatively small size of our study.

In our study, administration of creatine for two weeks prior to the exercise intervention was associated with a modest increase in thigh CSA that approached a level of statistical significance (p = 0.06 vs. placebo). Likewise, subjects in the creatine group had a significantly greater increase in LBM following exercise training (p = 0.01 vs. placebo). Since these changes were not accompanied by increases in muscle strength or improvement in indices of muscle fatigue or mitochondrial energy metabolism, it is conceivable that the increases in CSA and LBM could be explained by increases in muscle water content, rather than functional muscle mass. Indeed, increases in muscle water content during creatine treatment have been reported in prior studies [52]. An increase in water content in muscle may precede increases in muscle protein synthesis, which may confer functional (fitness) and physiological (e.g. glucose disposal) benefits with a longer period of follow-up. Moreover, increasing muscle size may be considered to be desirable in persons using creatine and exercise for aesthetic purposes.

All subjects showed robust increases in strength in eight important muscle groups following 12 weeks of PRT. These results provide further evidence of the beneficial effects of PRT in individuals with HIV infection, as has been reported previously in smaller studies [2]–[10]. In addition, the rate of PCr recovery following a 15-second MVC improved in the group as a whole. At baseline, average PCr recovery rate (T_1/2_) was 29 seconds, which is approximately 24% slower than those of healthy young and old adults studied with the same apparatus and using comparable methodology [24]. Following training, PCr recovery rates approached those in healthy untrained adults (T_½_≈23 seconds[24]). Because PCr recovery under the conditions applied in the present study reflects the capacity for oxidative phosphorylation, these data suggest that PRT improved mitochondrial energy metabolism in patients with HIV infection. Overall, these benefits provide further evidence that persons with HIV infection can adapt to a demanding exercise-training program.

Because HIV-infected persons on ART are at risk of developing metabolic and morphologic abnormalities, including peripheral lipoatrophy [53], we examined the effects of creatine supplementation and PRT on fat distribution and fasting lipids and glucose homeostasis. The observed preservation of subcutaneous fat during PRT suggests that this type of exercise did not promote or exacerbate lipoatrophy. Overall, we saw no evidence of improvement in metabolic and morphologic outcomes with either creatine supplementation or exercise. Because renal function is impaired in some patients with HIV infection [54], clinicians should be aware of the possibility that elevations in serum creatinine may occur in patients using creatine supplementation.

In summary, in this randomized, placebo-controlled trial of the effects of progressive resistance training with and without creatine supplementation in HIV-positive subjects, significant gains in strength were achieved with 12 weeks of PRT with no further benefit from creatine. The medical use of creatine supplementation in this population may be limited to aesthetic purposes, rather than to improve functional capacity. However, the efficacy and safety of PRT demonstrates its potential therapeutic benefit in preventing or reversing muscle weakness.

## Supporting Information

Checklist S1 CONSORT Checklist
(0.06 MB DOC)

Protocol S1 Trial Protocol
(0.10 MB DOC)
