# Supplementary material for: Creatine Fails to Augment the Benefits from Resistance Training in Patients with HIV Infection: A Randomized, Double-Blind, Placebo-Controlled Study
Source: PLoS One. 2009 Feb 26;4(2):e4605. doi: 10.1371/journal.pone.0004605 (PMC2646129; doi:10.1371/journal.pone.0004605)
Supplement: Protocol S1 — Trial Protocol (0.10 MB DOC) [file pone.0004605.s002.doc]

# ERGOGENIC EFFECTS OF CREATINE SUPPLEMENTATION

# CHR PROTOCOL H-1080-16219

**STUDY AIM AND PURPOSE:**

_ To test the hypothesis that short-term administration of creatine monohydrate improves skeletal muscle mass and strength, as well as muscle metabolic and performance responses to exercise, in patients with HIV infection.

_ To test the hypothesis that chronic administration of creatine augments the functional benefits of progressive resistance training related to skeletal muscle mass and strength, as well as muscle metabolic and performance responses to exercise.

_ To evaluate the effects of creatine on systemic metabolic, immunologic, and virologic parameters.

**2. BACKGROUND**

As the focus of HIV management shifts from acute to long-term care, loss of lean body mass (LBM), in particular skeletal muscle, is emerging as one of the most disabling aspects of chronic HIV infection. Although progressive resistance exercise has been associated with marked improvements in weight, LBM, and strength in subjects with HIV infection (1-4), initiating and adhering to an exercise regimen that is sufficiently strenuous to result in these improvements is difficult. Creatine monophosphate, which is marketed as a dietary supplement, has gained popularity in the HIV community, as well as the athletic community as a whole, on the basis of widespread belief that it can enhance athletic performance (5,6). Anecdotal reports suggesting that increases in strength, endurance, and muscle mass are associated with creatine supplementation have motivated individuals to return to the gym and adhere to fitness programs they might have otherwise abandoned (6,7). These reports indicate that trainers and coaches, in particular, have commented favorably on creatine’s apparent potency and positive impact on adherence to exercise regimens.

Creatine (N-[aminoiminomethyl]-N-methyl glycine) is found primarily in skeletal muscle, where it exists in both a free and phosphorylated form, phosphocreatine (PCr). Phosphocreatine is an important energy source in muscle, by virtue of the transfer of its phosphate group to adenosine diphosphate (ADP) to form adenosine triphosphate (ATP), a reaction that is catalyzed by the enyzme creatine kinase. This process of energy regeneration is particularly important during short duration high-intensity physical activity, as ATP is the source of energy for muscle contraction, and intramuscular stores of ATP are limited. Creatine supplementation in healthy subjects has been shown to increase total body mass (8-10), although these findings may reflect water retention. Unfortunately, only a small number of studies have been performed to look at the effect of creatine together with exercise training on LBM (8-12). Although some of these studies suggest that creatine therapy increases LBM, concomitant changes in hydration status may also have affected these results. Further studies are needed to determine the precise effect of chronic creatine supplementation together with exercise training on LBM and to evaluate whether measured increases in LBM reflect increased functional tissue or merely increased hydration of LBM.

While the effects of creatine supplementation in healthy subjects are unclear, studies of populations with initial deficits in muscle function generally indicate a beneficial effect. For example, the effects of creatine supplementation on PCr availability and resynthesis rates appear to be greater in older subjects (13). Subjects with mitochondrial cytopathies exhibited an 11% increase in performance during non-ischemic dorsiflexion exercise following 3 weeks of creatine supplementation (14). Ten days of creatine supplementation in subjects with chronic heart failure resulted in a 12% increase in muscle [PCr] (by biopsy) and significant increases in 1- and 2-legged exercise performance (15).

1. **SIGNIFICANCE**

Increased PCr availability and improved physical exercise performance with creatine supplementation form the basis for creatine use as an ergogenic aid in the community. However, the scientific evidence for its efficacy has not been clearly established. Not all studies demonstrate a positive effect of creatine supplementation, and careful randomized placebo-controlled studies are necessary to evaluate its safety and efficacy.

As concern about potential metabolic toxicities of highly active antiretroviral therapy (HAART) mounts, there is increasing interest in nonpharmacologic approaches to management of the metabolic complications of HIV infection and its therapies. Because resistance exercise is known to improve insulin resistance and lipid profiles and reduce abdominal fat, in addition to increasing muscle mass, patients in the current treatment era are frequently encouraged to undertake such training. Laboratory studies consistently demonstrate the positive effects of exercise, but a remaining challenge is to identify ways of encouraging patients to not only initiate but also adhere to an exercise regimen. If creatine supplementation can help in this latter effort, it could be a useful nonpharmacologic means of assisting with efforts to not only increase muscle mass but also manage the modern metabolic complications of HIV infection.

1. **METHODS**

**A. General Study Design:** This is a randomized, placebo-controlled study to evaluate the effect of creatine supplementation on skeletal muscle size and function (i.e., strength, energy metabolism, work capacity, fatigue); whole-body exercise performance; body composition; systemic metabolic parameters; immune function; and viral replication. This study is also designed to determine whether creatine supplementation augments the functional benefit derived from progressive resistance exercise. Forty HIV+ subjects will be randomly assigned, on a 1:1 basis, to receive creatine monohydrate or placebo for a period of 14 days, followed by a 12-week program of progressive resistance exercise training during which administration of creatine monohydrate or placebo will continue.

**B. Methods of Data Analysis:** Sample size calculations and methods of data analysis were prepared in consultation with Dr. Peter Bacchetti, biostatistician for the General Clinical Research Center (GCRC) at San Francisco General Hospital (SFGH). Intent-to-treat analyses will include all subjects who provide outcome data. Within each group, the one-sample t-test will be used for change measures that appear to be normally distributed, and the Wilcoxon signed-rank test will be used otherwise. Comparisons between the creatine and placebo groups will be by two-sample t-test or by Wilcoxon rank-sum test.

**C. Subject Selection:**

**1. Who and why:** HIV-positive adults who are currently clinically stable will be recruited for study. We anticipate that such individuals will be able to engage in a sufficiently rigorous exercise training regimen to enable us to determine whether creatine supplementation is an effective ergogenic aid.

**2. Total number:**  A sample size of 20 per group will allow for detection of standardized effect size of 0.9 with 80% power.

**3. Inclusion criteria**: HIV-positive subjects must be on optimized antiretroviral regimens, as determined by their primary medical providers, and plan to remain so during the study. Men and women on hormone replacement therapy and women using hormonal contraceptives must have been on stable regimens for the preceding 6 months and plan to continue on such treatment throughout the study period.

**4. Exclusion criteria:** Serum creatinine > 1.5 mg/dl or clinical evidence of renal disease or prior kidney transplant; creatine kinase (CK) > 1.5 times the upper limit of normal (ULN); hemoglobin < 8.5 g/dl; AST, ALT, or LDH ≥5 X ULN; uncontrolled diarrhea (>6 stools per day); impaired oral intake; persistent nausea or vomiting; untreated hypogonadism; pharmacologic use of growth hormone, testosterone, oxandrolone, nandrolone decanoate, oxymetholone, or other oral, injectable, or transdermal anabolic steroids, androstenedione, or dehydroepiandrosterone (DHEA) within the preceding 6 months (subjects with documented hypogonadism on stable testosterone replacement, defined as a dose <300 mg q2 weeks for the preceding 6 months, will be allowed to enroll); use of glucocorticoids, megestrol acetate, creatine monohydrate, cytokine inhibitors (thalidomide, pentoxifylline, ketotifen), drugs known to adversely affect renal function, cytokines, parenteral or tube feeding, or initiation of treatment for a systemic infection within 30 days prior to enrollment; history of angina, coronary heart disease, or congestive heart failure; current pregnancy or lactation or plans to become pregnant. Because vegetarians are known to have lower intramuscular concentrations of creatine and therefore may experience a much greater relative increase in muscle creatine levels, we will exclude such individuals from this study.

**D. Subject Recruitment:** Recruitment will be by advertisement, ongoing interaction with local physicians and AIDS organizations such as the Community Consortium, and word of mouth.

**E. Consent:** The purposes of the study will be explained to the subjects by Drs. Schambelan, Kent-Braun, Johansen, Sakkas, Mulligan, or Lo or their associates, and those who are interested be invited to sign a consent form at the time of the screening visit.

#### **F. Experimental protocol:**

**1. Screening:** The assessment of eligibility will include a medical history targeted to HIV and cardiovascular disease; physical examination; exercise, weight, and antiretroviral history; electrocardiogram; current medication inventory; and nutritional assessment by 24-hour recall. Blood samples will be collected for standard chemistries and hematology to exclude subjects with any significant laboratory abnormality as listed above.

**2. Baseline assessments:** Eligible subjects will undergo baseline testing that includes measurements of muscle cross-sectional area and isometric strength, fatigue and metabolism; dynamic strength of knee and ankle flexors and extensors; habitual physical activity; and total and regional body composition. Blood samples will be collected for measurement of lipids and lipoproteins, glucoregulatory, gonadal, and adrenal hormones, viral load, and indices of immune function. Subjects will prepare a 3-day food intake diary to quantitate baseline energy and macronutrient intake and meat consumption.

**3. Randomization:** Subjects will then be randomized, in a 1:1 fashion, to receive either creatine or placebo, following the dosing regimen described below. Between the 12^th^ and 14^th^ day after randomization, subjects will repeat all of the measurements performed at baseline except for physical activity and food intake. All subjects will then begin a 12-week progressive resistance exercise program, consisting of 3 training sessions per week. Subjects will remain on their assigned oral study regimens (creatine or placebo) during these 12 weeks.

**4. Creatine Dosing:** Although current dosing regimens for creatine supplementation are known to vary widely in the community, we have selected a dosing regimen based on loading, maintenance, and rest cycles used by creatine manufacturers and experts in the use of creatine for athletic training. The loading dose is a total of 20 g pure creatine monohydrate or placebo in capsules, taken in four divided doses daily with a non-caffeinated solution containing 20 g of simple carbohydrate. This regimen will be followed for 5 consecutive days, after which subjects will begin maintenance dosing of 4.8 g daily. Maintenance dosing will continue for five weeks, after which this cycle of loading and maintenance will begin again.

**5. Procedures:** Unless noted otherwise, all procedures will be performed in the GCRC at SFGH.

*a) Habitual activity:*  Daily activity will be measured with a 3-dimensional accelerometer (16-18). Each subject will be instructed in the use of the monitor, and data will be acquired for 7 consecutive days. The monitor will be worn in a pack snugly fit around the waist during all waking hours, except time spent in bathing or swimming. Each subject will also keep a simple written record of physical activity for those 7 days.

*b) Muscle size and function:* Measurements of muscle function will be performed using magnetic resonance spectroscopy (MRS), and cross-sectional muscle area by magnetic resonance imaging (MRI), at the San Francisco VAMC. Maximal isometric contraction force (MVC) will be measured in the unfatigued dorsiflexor muscles of all subjects. Each subject will be placed in the leg exercise apparatus with the foot securely strapped to the force platform. The apparatus will then be loaded into the magnet, and the subject positioned comfortably in an upright position. Three MVCs lasting 3-4 seconds each will be performed, separated by 1 minute rest. Two exercise protocols will then be performed. The first, to assess the rate of PCr recovery (an index of oxidative capacity), will consist of a 15 sec MVC. The second exercise protocol, used to quantitate initial PCr hydrolysis rate, initial rate of fatigue, total work capacity and total fatigue, will consist of 60 consecutive MVC's, one every 2 seconds. Contractions will be paced using a pre-recorded cassette tape, and subjects will be given strong verbal encouragement throughout each exercise. Both protocols will be performed on the same day, with a 20 minute rest period between them.

Fat-free muscle and fat cross sectional areas (CSA) of the lower leg, quadriceps, and gluteal region will be determined using MRI (19). A total of 33 transverse slices will be acquired in an interleaved fashion (slice thickness = 4 mm, echo time = 14 ms, repetition time = 510 ms, flip angle = 70°, field of view = 210 mm, 256 X 256 matrix, 2 acquisitions). The slice in which the anterior compartment CSA is largest will then be analyzed for fat-free and fat-containing areas. Quadriceps and gluteal muscle and fat CSA will be measured in a similar fashion. Ten-millimeter thick image slices will be collected from the head of the femur to the knee joint. The slice with the largest muscle CSA and the two adjacent slices will be used for analysis, as described above. The time required to complete both the MRS and MRI testing is 3-4 hours.

*c) Body composition:* Whole-body and regional fat and lean body mass will be measured by dual-energy X-ray absorptiometry (DEXA). Subjects lie on a padded scanning table while wearing a standard hospital gown, underwear, and pajama bottoms that contain no snaps or other material that may interfere with attenuation. The procedure takes approximately 20 minutes for most subjects.

*d) Total body water* will be measured by deuterium oxide dilution. After collection of a baseline plasma sample, subjects will drink 10 g of deuterium oxide (99.9 atom %). The drinking vessel will be rinsed with three successive volumes of 20 ml each of tap water, such that the total fluid intake will be 70 ml. During the following three hours the subject will sit or rest, avoiding any strenuous activity. After three hours a second plasma sample will be collected.

*e) Anthropometry*: Waist, hip, neck, chest, thigh, and midarm circumferences; and subscapular, triceps, abdominal, and thigh skinfold thicknesses will be measured in triplicate by a trained dietitian (20).

*f) Resistance exercise training:* Following testing at the end of the baseline period (week 2), all subjects will begin 12 weeks of progressive resistance exercise training. Training will be performed under the guidance of an American College of Sports Medicine (ACSM) certified Exercise Specialist. Prior to starting any of the exercises, all subjects will warm up for 5 minutes on a cycle ergometer and all muscle groups to be exercised will be stretched 3 times for 20 seconds each. Initial muscle strength will be measured and peak isometric force and dynamic torque will be used to develop a program for training of the muscle groups. Dynamic training will begin with 2 sets of 10 repetitions at 70% 1 repetition maximum (RM), which is the maximum amount of weight that can be lifted through a joint’s range of motion, for the first 2 weeks, and then will progress to 2 sets of 10 repetitions at 85% 1RM for the next 10 weeks. All subjects will train 3 times per week, and each training session will be supervised. The protocols to be used are similar to those used in other strength training studies (21,22) and apply the principles of gradual progression, overload, and specificity of training known to be necessary to achieve muscle strength improvements (23-26). Subjects will be re-tested on the training equipment every 2 weeks to reassess maximal strength levels, and the training intensities will be adjusted to reflect any strength changes.

*g) Follow-up assessments:* After initiation of exercise training, subjects will undergo monthly assessment of weight, body composition by DEXA and anthropometry. Blood samples will also be collected at these visits for safety monitoring. At the end of the 12-week training period (week 14 of the study) subjects will repeat all of the assessments performed before initiation of exercise, including assessment of habitual physical activity and food intake.

###

### Safety Monitoring: Because increases in serum creatinine and CK levels have been reported in some people using creatine (7,27), these parameters will be monitored every other week. If serum creatinine levels increase to above 1.8 mg/dl or CK increases to > 450 U/liter in men or 400 in women (ULN 226 in men and 140 in women), study medication will be held for one week. If, after one week, creatinine and/or CK levels have returned to baseline levels, study medication will be resumed at half of the initial dosing level and creatinine and/or CK levels monitored weekly. If creatinine and/or CK levels again exceed the aforementioned values on this reduced dosing regimen, study drug will be discontinued and the subject followed on a biweekly basis until these levels return to within acceptable limits.

**G. Risks and Discomforts**

**1. Side Effects of Medications:** Serum creatinine levels may increase slightly with creatine therapy, although there have been no reports of overt renal dysfunction except for one case in which the subject had underlying nephrotic syndrome and glomerulosclerosis (28). Transient muscle cramps and elevation in circulating creatine kinase levels have also been reported (7,27). Some subjects taking creatine supplements have complained of nausea, stomach upset, dizziness or weakness, loose stools, or diarrhea at daily serving sizes greater than 5 grams (6,7). As described above, we will monitor serum creatinine and CK levels and reduce or discontinue dosing with study medication if warranted.

**2. Exercise and Strength Testing:**  Resistance exercise and muscle strength testing may result in muscle fatigue or soreness. Joint discomfort may be experienced if the exercises are not performed correctly. To minimize these risks, the starting resistance used will be individualized according to strength and tolerance and progression of the program will be gradual. In addition, all training will be monitored by an ACSM certified Exercise Specialist.

**3. Magnetic Resonance Spectroscopy/Imaging:** There are no significant risks associated with the MRS measurements, aside from the possibility of a magnetic object being pulled into the magnet. However, precautions have been taken to prevent such an event from happening; loose metal objects such as jewelry, pocket knives, or key chains, are not allowed in the MRS room. The subject may become fatigued from prolonged sitting in the magnet, and relatively small amounts of discomfort might be experienced when the subject is asked to exercise his/her limb for a prolonged period of time. Some soreness in the muscles may appear after muscle testing, but this will be slight, will not impede normal daily activity, and will subside within a few days. For the MRI portion of the study, subjects may be bothered by feelings of claustrophobia and by the loud noise during this part of the study. Temporary hearing loss has been reported, so subjects will be asked to wear ear plugs.

**4. Radiation Exposure***:* Exposure from DEXA is less than 5 mrem per measurement, which is less than 1% of the exposure from a chest X-ray. The stable isotope ^2^H (deuterium) contains no radioactivity and has no recognized adverse effects.

**5. Venipuncture:** Venipuncture and placement of intravenous lines may result in a hematoma, which can be painful but carries no significant risks. Infections from IV lines rarely occur from such brief placements. However, any signs of inflammation will lead to removal of the catheter and treatment with heat and elevation.

**6. Phlebotomy:** The total amount of blood drawn will not exceed 200 cc.

**H. Treatment and Compensation for Injury:** If a subject is injured as a result of being in this study, treatment will be available. The cost of such treatment may be covered by the University of California. Subjects will be informed that they may obtain further information by calling the Committee on Human Research.

**I. Alternatives:** Subjects may choose not to participate in the study.

**J. Costs to the Subject:** There will be no charges to the subject. The costs of the study medication and all tests required for the study will be covered by the study. Transportation costs to and from the VAMC will also be reimbursed or covered by taxi vouchers.

**K. Reimbursement of Subjects:** Subjects will be reimbursed $1,000 for completing the study. Subjects who do not complete the full study will be reimbursed $200 for each major assessment (baseline and week 2).

**L. Confidentiality of Records:** Participation in research may involve loss of privacy. The subjects' research records will be handled as confidentially as is possible within the law. All records will be coded and stored in the office of the PI. Copies of the signed consent forms are kept by the GCRC, the subjects, and the PI. Copies of the subjects’ medical records may be reviewed by representatives of the Food and Drug Administration. No individual identities will be used in any reports or publications resulting from this investigation.

**5. QUALIFICATIONS OF INVESTIGATORS:** Over the past six years, Drs. Mulligan and Schambelan have conducted numerous studies of anabolic and anticytokine therapies and their metabolic effects in HIV-infected individuals with weight loss, as well as studies of body composition and energy metabolism in groups of men and women with HIV infection. Dr. Kent-Braun has performed numerous studies of muscle metabolism and function in subjects with a variety of chronic catabolic conditions or neuromuscular disorders.

**6. BIBLIOGRAPHY:** attached.

##### Attachment 1. Bibliography

1. Spence DW, Galantino MLA, Mossberg KA, Zimmerman SO. Progressive resistance exercise: effect on muscle function and anthropometry of a select AIDS population. Arch Phys Med Rehabil 1990; 71:644-648.
2. Rigsby LW, Dishman RK, Jackson AW, Maclean GS, Raven PB. Effects of exercise training on men seropositive for the human immunodeficiency virus-1. Med Sci Sports Exerc 1992; 24:6-12.
3. Macarthur RD, Levine SD, Birk TJ. Supervised exercise training improves cardiopulmonary fitness in HIV-infected persons. Med Sci Sports Exerc 1993; 25:684-688.
4. Roubenoff R, Suri J, Raymond J, Fauntleroy J, Gorbach S. Feasibility of increasing lean body mass in HIV-infected adults using progressive resistance training. Nutrition. 1997; 13:271 Abstract.
5. Anonymous. Creatine and androstenedione - two "dietary supplements". The Medical Letter 1998; 40:105-106.
6. Sahelian R, Tuttle D. Creatine. Nature's muscle builder. Garden City Park, NY: Avery Publishing Group, 1997.
7. Sahelian R. Creatine. 1998.
8. Earnest CP, Snell PG, Rodriguez R, Almada AL, Mitchell TL. The effect of creatine monohydrate ingestion on anaerobic power indices, muscular strength and body composition. Acta Physiol Scand 1995; 153:207-209.
9. Krieder R, Ferreira M, Wilson M, et al. Effects of creatine supplementation on body composition, strength, and sprint performance.. Med Sci Sports Exerc 1998; 30:73-82.
10. Williams MH, Branch JD. Creatine supplementation and exercise performance: An update. J Am Coll Nutr 1998; 17:216-234.
11. Kirksey KB, Warren BJ, Stone MH, Stone MR, Johnson RL. The effects of six weeks of creatine monohydrate supplementation in male and female track athletes. Med Sci Sports Exerc. 1997; 29:S145 Abstract.
12. Stout JR, Eckerson J, Noonan D, Moore G, Cullen D. The effects of a supplement designed to augment creatine uptake on exercise performance and fat free mass in football players. Med Sci Sports Exerc. 1997; 29:S251 Abstract.
13. Smith SA, Montain SJ, Matott RP, Zientara GP, Jolesz FA, Fielding RA. Creatine supplementation and age influence muscle metabolism during exercise. J Appl Physiol 1998; 85:1349-1356.
14. Tarnopolsky MA, Roy BD, MacDonald JR. A randomized, controlled trial of creatine monohydrate in patients with mitochondrial cytopathies. Muscle and Nerve 1997; 20:1502-1509.
15. Gordon A, Hultman E, Kaijser L, et al. Creatine supplementation in chronic heart failure increases skeletal muscle creatine phosphate and muscle performance.. Cardio Res 1995; 30:413-418.
16. Matthews CE, Freedson PS. Field trial of a three-dimensional activity monitor: Comparison with self report. Med Sci Sports Exerc 1995; 27:1071-1078.
17. Miller DJ, Freedson PS, Kline GM. Comparison of activity levesl using the Caltrac accelerometer and five questionnaires. Med Sci Sports Exerc 1994; 26:376-382.
18. Ng AV, Kent-Braun JA. Quanititation of lower physical activity in persons with multiple sclerosis. Med Sci Sports Exerc 1997; 29:517-523.
19. Kent-Braun JA, Ng AV, Castro M, Dudley GA, Miller RG. Strength, skeletal muscle composition and enzyme activity in multiple sclerosis. J Appl Physiol 1997; 83:1998-2004.
20. Lohman TG, Roche AF, Martorell R. Anthropometric standardization reference manual. Human Kinetics Books, 1988.
21. Fiatarone M, Marks E, Ryan N, Meredith C, Lipsitz L, Evans W. High-intensity strength training in nonagenarians. J Am Med Assoc 1990; 263:3029-3034.
22. Frontera W, Meredith C, O'Reilly K, Knuttgen H, Evans W. Strength conditioning in older men: Skeletal muscle hypertrophy and improved function. J Appl Physiol 1988; 64:1038-1044.
23. Altha J. Strengthening muscle. Philadelphia: The Franklin Institute Press, 1981.
24. Coyle E, Feiring D, Rotkis T, et al. Specificity of power improvements through slow and fast isokinetic training.. J Appl Physiol 1981; 51:1437-1442.
25. Lesmes G, Costill D, Coyle E, Fink W. Muscle strength and power changes during maximal isokinetic training. Med Sci Sports Exerc 1978; 10:266-269.
26. Moffatt R, Cucuzzo N. Strength consideration for exercise prescription. 2nd ed. Philadelphia: Lea & Febiger, 1993.
27. Almada A, Mitchell T, Earnest C. Impact of chronic creatine supplementation on serum enzyme concentrations. FASEB J 1996; 10:A791 Abstract.
28. Pritchard NR, Kalra PA. Renal dysfunction accompanying oral creatine supplements. Lancet 1998; 351:1252

**Attachment 2. Study timetable**

|  | | *Creatine or placebo throughout study* | | | | | | | | |
| --- | --- | --- | --- | --- | --- | --- | --- | --- | --- | --- |
|  | | | | ***Progressive resistance exercise 3X weekly beginning at week 3*** | | | | | | |
|  | 0 | | Wk 2 | Wk 4 | Wk 6 | Wk 8 | wk 10 | Wk 12 | wk 14 |  |
| 1 Repetition Max | X | | X | X | X | X | X | X | X |  |
| MRI | X | | X |  |  |  |  |  | X |  |
| MRS | X | | X |  |  |  |  |  | X |  |
| Total body water | X | | X |  |  |  |  |  | X |  |
| Accelerometer | X | |  |  |  |  |  |  | X |  |
| DEXA | X | | X |  | X |  | X |  | X |  |
| Anthropometry | X | | X |  | X |  | X |  | X |  |
| 3-day food intake diaries | X | |  |  |  |  |  |  | X |  |
| Serum creatinine, CK | X | | X | X | X | X | X | X | X |  |
| Chemistries | X | | X |  | X |  | X |  | X |  |
| Hematology | X | | X |  | X |  | X |  | X |  |
| Hormones | X | | X |  |  |  |  |  | X |  |
| Virology | X | | X |  |  |  |  |  | X |  |
| Immunology | X | | X |  |  |  |  |  | X |  |

##### Attachment 3: Creatine dosing regimen

- Loading dose: 20 g/day pure creatine monohydrate powder or placebo in capsules (in four divided doses). Each dose will be taken together with a non-caffeinated solution containing 20 g of simple carbohydrate . This regimen will be followed for 5 consecutive days, after which patients will begin maintenance dosing.
- Maintenance dose: 4.8 g/day pure creatine monohydrate powder or placebo in capsules (in two divided doses), to be taken with a non-caffeinated carbohydrate solution as specified above.

|  | *Wks 1-2* | **Wks 3-4* | *Wks 5-6* | *Wks 7-8* | *Wks 9-10* | *Wks 11-12* | *Wks 13-14* |
| --- | --- | --- | --- | --- | --- | --- | --- |
| *CREATINE/ PLACEBO* |  |  |  |  |  |  |  |
| Load | X |  |  | X |  |  | X |
| Maintenance | X | X | X | X | X | X | X |

**** Exercise regimen begins and continues for 12 weeks***
